# Suitability of Endovascular Materials for Physician-Modified Fenestrated Endografts in Urgent Juxtarenal and Pararenal Aortic Pathologies

**DOI:** 10.3390/jcm14144830

**Published:** 2025-07-08

**Authors:** Mario Lescan, Aleksandar Dimov, Davide Turchino, Alexandru Toma, Johannes Scheumann, Tim Berger, Maximilian Kreibich, Roman Gottardi, Martin Czerny, Stoyan Kondov

**Affiliations:** 1 Department of Cardiovascular Surgery, Heart Centre Freiburg University, 79106 Freiburg, Germany; aleksandar.dimov@uniklinik-freiburg.de (A.D.); alexandru.toma@uniklinik-freiburg.de (A.T.); johannes.scheumann@uniklinik-freiburg.de (J.S.); tim.berger@uniklinik-freiburg.de (T.B.); maximilian.kreibich@uniklinik-freiburg.de (M.K.); roman.gottardi@uniklinik-freiburg.de (R.G.); martin.czerny@uniklinik-freiburg.de (M.C.); stoyan.kondov@uniklinik-freiburg.de (S.K.); 2 Faculty of Medicine, Albert Ludwig’s University of Freiburg, 79110 Freiburg, Germany; 3 Vascular Surgery Unit, Department of Public Health, University Federico II of Naples, 80131 Naples, Italy; dott.turchino@gmail.com

**Keywords:** physician-modified, endograft, juxtarenal, pararenal, aneurysm, AAA

## Abstract

**Background/Objectives**: Physician-modified endografts (PMEGs) have emerged as a treatment option for complex aortic pathologies. Uncertainty remains regarding the modification techniques and the most suitable materials for customization of fenestrated endografts. The aim of this study was to evaluate CE-marked endovascular aortic repair (EVAR) devices and suitable materials for device modification in PMEGs for juxtarenal and pararenal aortic pathologies. **Methods**: This single-center observational study included patients treated with the physician-modified TREO (Terumo Aortic, Inchinnan, UK) device between April and December 2024. All patients had aortic ruptures or symptomatic aneurysms and unfavorable anatomy or severe comorbidities, making standard EVAR and open repair unsuitable. Procedural data were recorded and analyzed, including in-hospital outcomes. The “wire visibility” and “sheath–wire contrast” of endografts were assessed under fluoroscopy, and different resheathing techniques were compared. **Results**: Technical success was achieved in all five patients. The number of fenestrations per patient was 2.6 (range: 1–4). In one patient (1/5), type Ib and type IIIc endoleaks were observed postoperatively, requiring reintervention. No in-hospital mortality occurred. The ICU and hospital stay were 24 h (range: 18–40 h) and 8 days (range: 6–20 days), respectively. Moreover, the One SNARE wire was identified as the wire with the highest “wire visibility”, and Endurant II showed the best “sheath–wire contrast”. Resheathing with the dedicated crimping device was superior to the tape-assisted method. **Conclusions**: The TREO platform, in synergy with suitable additional materials, offers a viable solution for urgent aortic pathologies requiring PMEGs. Continued refinement of materials and procedural standardization could enhance the long-term outcome.

## 1. Introduction

Abdominal aortic aneurysms (AAAs) are a common vascular condition and carry a significant risk of rupture, particularly as the aneurysm diameter increases. Most patients remain asymptomatic until the aneurysm reaches a critical size, at which point the risk of fatal rupture becomes substantial [1]. Rupture is associated with high mortality rates, estimated at 60–80%, with a considerable number of deaths occurring both before hospital admission and during in-hospital treatment [2]. Therefore, current European guidelines recommend elective repair in men once the aneurysm reaches 55 mm in diameter, or 50 mm in women, as well as in cases of rapid aneurysm growth or the onset of symptoms [3,4].

Endovascular aortic repair (EVAR) is equally recognized alongside open aortic repair (OAR) as the gold standard for treating infrarenal abdominal aortic aneurysms in both elective and urgent settings [3,4]. Its minimally invasive nature makes it a preferred option for patients with severe comorbidities and advanced age in comparison to the open repair. However, anatomical challenges, such as the absence of a healthy landing zone below the renal artery origins, can limit the feasibility of EVAR, while OAR remains viable through infrarenal, transrenal, or suprarenal clamping.

In elective cases, extending the proximal landing zone above the renal artery origins can also be achieved by applying custom-made fenestrated endografts. However, the production time for these devices often spans several weeks, during which there is a risk of aneurysm rupture [5]. Chimney techniques are approved for treating juxtarenal or no-neck aneurysms. Still, they should be limited to renal arteries due to the significant risk of gutter endoleaks, particularly in cases involving more than two chimneys or anatomically unsuitable conditions [6]. Off-the-shelf branched endovascular aortic repair (BEVAR) devices offer another alternative but are associated with an increased risk of paraplegia due to the extensive proximal coverage required [7].

In the absence of off-the-shelf fenestrated endovascular aortic repair (FEVAR) options, patients requiring urgent repair and presenting with severe comorbidities demand an alternative minimally invasive, “ready-to-use” treatment approach. Recently, physician-modified endografts (PMEG) have gained prominence for meeting these requirements. This method necessitates meticulous planning, advanced manual skills, in-depth knowledge of materials, and endograft implantation.

The aim of this study was to evaluate the clinical use of the TREO (Terumo Aortic, Inchinnan, UK) EVAR device for creating PMEG endografts and to assess the suitability of various endovascular disposables to optimize the technique.

## 2. Materials and Methods

### 2.1. Study Design and Population

In this single-center observational study from 06/2024 to 12/2024, all consecutive patients treated for urgent aortic pathologies with TREO (Terumo Aortic, Inchinnan, UK) PMEG endografts were included. Patients who were treated with custom-made TREO FEVAR grafts (n = 2) or Endurant II (Medtronic, Santa Rosa, CA, USA) PMEG (n = 1) during the study period were excluded from the study. All inclusion and exclusion criteria are listed in Table 1. The urgent pathologies included aortic rupture, symptomatic aneurysms, and type Ia endoleaks with diameter progression during the follow-up. Moreover, all patients were discussed in the center’s weekly interdisciplinary aortic team conference (comprising interventional radiologists, angiologists, and vascular surgeons) or ad hoc conferences to assess the operability of the OAR and anatomical feasibility of EVAR. In cases of their absence, the indication for PMEG was determined. Patients were informed about the procedure, the unclear mid- and long-term durability of the PMEG devices, and the alternative treatment options. All patients provided consent to the operation. The ethical review board approved the study and waived the patient’s consent due to the study’s observational nature.

### 2.2. PMEG Planning

PMEG planning was conducted using Endosize version 3.2.1 (Therenva, Rennes, France). This dedicated planning software enables precise measurements along the automatically generated centerline and facilitates the assessment of the clock positions of target vessels. The presence and quality of the proximal sealing zone determined the number of fenestrations. The targeted healthy proximal landing zone length was 20 mm, with a planned oversizing of 15–20%. A healthy landing zone was defined as an area free of circumferential thrombus or calcification and by the absence of a bubble neck, tapered neck, or an aortic diameter exceeding 31 mm. The fenestration diameters were matched to the corresponding target vessel diameters and were designed to be equal in size to the designated bridging stent grafts (BSG; 5–8 mm).

### 2.3. Materials and Device Modification

**Figure 1:**
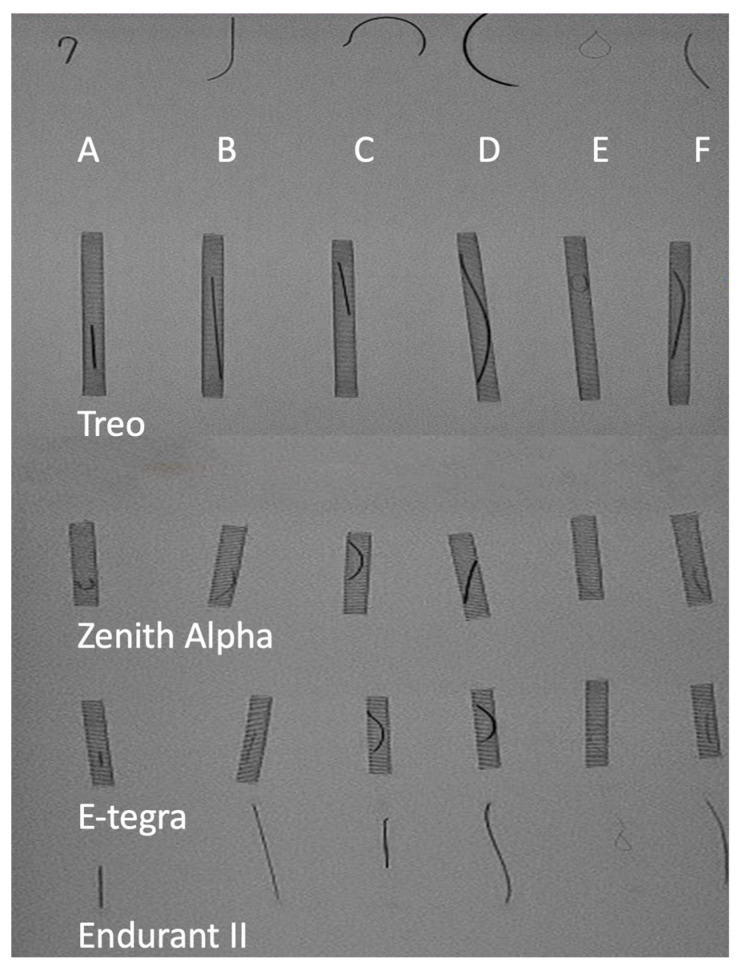
Fluoroscopy of various wires ((A) Rosenwire; (B) Radiofocus guidewire M; (C) One SNARE; (D) Lunderquist; (E) En-Snare; (F) Amplat super stiff) accommodated in commercially available EVAR system sheaths (TREO, Zenith Alpha, E-tegra, Endurant II).

The decision to use the TREO endograft was based on prior experience with its performance in standard EVAR procedures at our center [8,9]. The TREO device is available in two designs, namely as a bifurcated or tubular EVAR configuration with proximal suprarenal and infrarenal fixation [9]. It is enclosed within an 18–19 Fr sheath reinforced with a metallic spiral. The sheath was tested preoperatively to ensure clear visibility of the fenestrations and markers required for PMEG modifications (Figure 1). Therefore, different wires were placed into tubes previously cut from the introduction sheaths of four frequently used infrarenal EVAR systems, namely TREO, Zenith Alpha (Cook Medical, Bloomington, IN, USA), E-tegra (Artivion, Hechingen, Germany), and Endurant II (Medtronic, Santa Rosa, CA, USA), and then fluoroscopy was performed in the hybrid room. The assessment of the “wire visibility” and the “sheath–wire-contrast” parameters was performed independently by four vascular surgeons. During the endograft preparation, a partial deployment was performed approximately 1 cm below the lowest fenestration (Video S1).

The clock positions of the endograft were marked using a sterile flow-master at the 0, 3:00, 6:00, and 9:00 o’clock positions. Straight wire markers (One SNARE, Merit Medical, South Jordan, UT, USA) were sutured to the material at the 0 and 6:00 positions using 5-0 Prolene (New Brunswick, NJ, USA) in a continuous suture pattern. Fenestrations were created at the predefined clock positions and distances from the proximal endograft edge (proximal landing zone) using a cautery pen. They were subsequently reinforced with a gooseneck snare loop (One SNARE, Merit Medical, South Jordan, UT, USA), secured using a running 5-0 Prolene suture. Resheathing of the modified endograft was achieved using polyester tapes and tourniquets strategically placed in the midsection of the endograft ring stents. Constant pressure was applied to guide the sheath over the crimped endograft toward the tip of the deployment system (Video S1).

Bench testing for the resheathing of the device was performed to compare the resheathing of a standard TREO 24 mm bifurcated device in the described fashion (Video S1; polyester tapes and tourniquets) in comparison with a dedicated transaortic valve repair crimping device (MyVal, Meril, Vapi, Gujarat, India; Video S2). The time for the resheathing of the bifurcated device was measured.

### 2.4. Procedures

The endograft preparation and implantation were carried out by a consistent team of 2 experienced vascular surgeons, each with expertise in over 200 branched and fenestrated aortic repair procedures. The endograft was introduced into the femoral artery over a Lunderquist wire (Cook Medical, Bloomington, IN, USA) following a surgical cutdown. Orientation of the endograft toward the lower renal artery was achieved without axial C-arm rotation. Craniocaudal corrections were performed in all cases, ensuring precise alignment through the exact overlay of the anterior and posterior markers at the 0 and 6:00 o’clock positions. The deployment proceeded until the release of the contralateral limb in the bifurcated design, while the tube grafts were fully released. For the direct cannulation of the fenestrations, a steerable Destino Twist (Oscor, Palm Harbor, FL, USA) 7 Fr sheath was employed. Following successful cannulation of the target vessels, the bridging stent grafts (BSG) were deployed over a Rosenwire (Cook Medical, Bloomington, IN, USA) and subsequently flared with 8, 10, or 12 mm Powerflex balloons (Cordis, Santa Clara, CA, USA), selected according to the diameter of the BSG. The BSGs utilized in these procedures included BeGraft (Bentley, Hechingen, Germany), Advanta (Maquet, Rastatt, Germany), and VBX (Gore Medical, Newark, DE, USA). Ballooning of the EVAR graft was restricted to the distal endograft region and the overlap between the main body and iliac extensions, ensuring proper sealing without compromising the BSGs.

### 2.5. Variables and Analysis

All procedural and outcome parameters are presented in Table 1, Table 2, Table 3, Table 4 and Table 5 for clarity and comparison. Key procedural metrics, including procedural duration, average vessel cannulation time, and technical success rates, are detailed. Clinical outcomes, such as in-hospital mortality, target vessel patency, and the presence of endoleaks at discharge are also assessed. Continuous variables are reported as medians with ranges. Categorical variables are displayed as proportions or fractions, facilitating straightforward interpretation. The study adheres to the STROBE (Strengthening the Reporting of Observational Studies in Epidemiology) guidelines to ensure methodological rigor and transparency. The study was approved by the ethical committee of the University of Freiburg No. 23-1336-S1-retro.

## 3. Results

### 3.1. Bench Testing

Before clinical application, bench testing was conducted to evaluate the visualization of various disposables used for markers and fenestration reinforcements. The results of this testing are presented in Figure 1. The tips of the One SNARE (Merit Medical, South Jordan, UT, USA; Figure 1, “C”), Rosenwire (Cook Medical, Bloomington, IN, USA; Figure 1, “A”), and Lunderquist wire (Cook Medical, Bloomington, IN, USA; Figure 1, “D”) demonstrated superior visualization (wire visibility) compared to those of the Radiofocus Guidewire M (Terumo, Tokyo, Japan; Figure 1, “B”), EN Snare (Merit Medical, South Jordan, UT, USA; Figure 1, “E”), and Amplatz Super Stiff wire (Boston Scientific, Marlborough, MA, USA; Figure 1, “F”). The best wire visibility was found for the Lunderquist wire. Moreover, a good sheath–wire contrast was present in all tested sheaths, although the best sheath–wire contrast was in the Endurant II application system, which is due to the absence of a metallic support in the wall of this sheath. The interrater reliability for this testing was 1.0. Based on these findings, the smaller diameter of the One SNARE was deemed advantageous for the resheathing process, leading to its selection as the standard wire for this purpose, although its “wire visibility” was inferior to the Lunderquist wire. The bench testing for the device resheathing with the TAVR crimping system showed a faster resheathing (243 s vs. 97 s) in comparison with the tape-assisted technique used in this series.

### 3.2. Patient Demographics and Clinical Background

A total of five patients were treated with a physician-modified TREO endograft for urgent aortic pathologies during the study period. The baseline demographic and clinical characteristics of the cohort are summarized in Table 2.

The median patient age was 82 years (SD: 4.6), and there were three male patients. All five patients presented with a history of hypertension, while three had concurrent coronary artery disease (CAD) and atrial fibrillation (AF). Additional risk factors included prior abdominal surgery in one patient and a history of nicotine use in two other patients. The clinical indications for intervention are presented in Table 3. Two patients underwent treatment for ruptured aortic pathologies, with one undergoing treatment for a ruptured penetrating aortic ulcer (PAU) and the other for a ruptured infectious PAU. Type IA endoleaks, leading to aneurysm expansion, were observed in two patients. The remaining patient underwent urgent treatment for a symptomatic pararenal abdominal aortic aneurysm. Two patients from the cohort had previously undergone infrarenal aortic repair via endovascular procedures, with the reintervention dates recorded as March 2015 and July 2018, respectively. Cerebrospinal fluid (CSF) drainage was utilized in only one case.

### 3.3. Procedural Data and Clinical Outcomes

The intraprocedural characteristics for each patient are summarized in Table 4. The median procedural time was 120 min, with a range of 110 to 265 min. The number of fenestrations per patient ranged from 1 to 4, with an average of 2.6 fenestrations per case, and 13 fenestrations in total. Customizations of the TREO endograft included the modifications of three aortic tube grafts and two bifurcated devices, with the tube grafts being used for both failed EVAR cases. The use of bridging stent grafts varied accordingly. The utilized bridging stent grafts included the Gore Viabahn VBX (n = 8), Advanta V12 (n = 3), and Bentley BeGraft (n = 2).

The bridging stent grafts were selected based on the dimensions of the target vessels. The procedure with the longest operative time (350 min) was associated with the most complex anatomical configuration, involving four fenestrations. The bridging stent graft implantation times are presented in Table 5.

The median implantation time for the LRA was 8 min (range: 4–10), and 5 min (range: 2–5) for the RRA. The median implantation time for the SMA was 5 min (range: 3–13), while cannulation of the CT was necessary only in one single patient (10 min).

The post-procedural outcomes are summarized in Table 6. Technical success, defined as the successful deployment and cannulation of all target vessels without the angiographic presence of type I or III endoleak, was achieved in all five patients.

No major procedural complications, including target vessel occlusion or device malposition, were observed. Four patients demonstrated complete exclusion of the aneurysmal sac without evidence of endoleaks. One patient presented with a combined type IB and IIIc endoleak in the first postprocedural CTA follow-up, which necessitated reintervention 7 days after the primary procedure. The reintervention involved the relining of the bridging stent graft and the relining of the right iliac extension.

There were no in-hospital mortalities in this cohort. All patients were discharged alive after a median intensive care unit (ICU) stay of 24 h (range: 18–40 h). The total in-hospital stay had a median duration of 8 days (range: 6–20 days). The patient requiring the most complex intervention, which involved four fenestrations and multiple reinterventions, had the longest hospital stay of 20 days.

## 4. Discussion

Our findings emphasize the feasibility of the PMEG technique for managing acute juxta- and pararenal aortic pathologies. Technical success was achieved in all treated patients, with effective resolution of the underlying pathologies. Additionally, the pre-procedural identification of suitable disposables for marker placement and fenestration reinforcement proved invaluable, significantly enhancing visualization during in vivo implantation. The choice of the abdominal endograft plays a critical role in the success of PMEG modifications. In this study, the TREO device was selected due to prior experience and familiarity with its deployment system.

Several key criteria must be met by an EVAR graft to qualify for PMEG use. First, the deployment system should allow for retraction of the outer sheath via a pullback mechanism, a feature present in the most modern EVAR endografts. Second, the sheath material of the deployment system must demonstrate good X-ray permeability to enhance the visibility of additional markers. Third, the sheath material must exhibit sufficient durability to withstand potential damage during the resheathing process. Finally, the distance between the endograft struts and stent rows should provide adequate space to accommodate fenestrations in stent-free endograft fabric [10].

In comparison to the TREO deployment system sheath, Endurant II showed a better X-ray transparency with a better visibility of the sutured markers (Figure 1). This advantage, however, may be counterbalanced by the resheathing of the device. The metallic reinforcement of the TREO sheath protects the sheath surface from being roughened or damaged by the wire components of the endograft and shows more stability during the resheathing. Moreover, the TREO device offers more space between the stent rows for the accommodation of the fenestrations in a stent-free region in comparison to the Zenith Alpha (Cook Medical, Bloomington, IN, USA) [10].

Mid- and long-term outcomes of PMEG remain to be comprehensively evaluated. In contrast to European centers, the restricted availability of custom-made endografts in the United States in recent years has allowed for the generation of the first long-term PMEG results [11]. Starnes et al. [12] reported outcomes from 217 patients treated with various PMEG devices, demonstrating an aneurysm-related survival rate of 93% at five years and a freedom-from-reintervention rate of 60% over the same period. While the reintervention rate may initially seem high, only one late partial explant was required for distal graft thrombosis, and three ruptures occurred during follow-up in the third, fourth, and fifth years post-implantation. These findings are consistent with the 5-year outcomes reported by Chait et al. [13], who observed a 92% aorta-related survival rate in 156 patients treated with a modified Cook endograft. Asirwatham et al. [14] demonstrated a high technical success rate for the TREO PMEG device, even in patients with prior failed EVAR. Comparatively, custom-made fenestrated devices showed similar 1-year target vessel patency, endoleak rates, and survival; however, the reintervention rate was notably higher in the PMEG group (32% vs. 12%) [15]. A similar trend was noted by Sanders et al., who reported a 5-year reintervention rate of 37% for PMEG compared to 26% for custom-made FEVAR [16]. As already mentioned, in acute pathologies, custom-made endografts do not represent a viable alternative to PMEG due to the absence of immediate availability. Chimney aortic repair may provide one possible endovascular alternative for PMEG repair, with comparable early outcomes. However, this technique is associated with significant reinterventions in follow-up [11,17].

### Study Limitations

The main limitation of our series is the small patient cohort and the relatively short follow-up period. Moreover, only materials used in our department are evaluated in this study. More suitable endografts and modification materials for PMEG may exist. The idea of this work is to show which preparations should be carried out before the modifications of approved endografts and to present the results for frequently used endovascular materials.

## 5. Conclusions

The TREO endograft was shown to be a feasible option for creating physician-modified fenestrated endografts (PMEGs) in the treatment of urgent juxtarenal and pararenal aortic pathologies when open repair or custom-made FEVAR are not an option. Pre-procedural planning and the selection of appropriate materials for marker placement and fenestration reinforcement are critical in improving procedural accuracy and safety. Although the TREO device demonstrated several advantages in terms of compatibility and deployment, further improvements in material selection (e.g., dedicated resheathing devices) and further standardization of the PMEG process are necessary to enhance device durability.

## Figures and Tables

**Table 1:** Study inclusion and exclusion criteria.

Inclusion Criteria	Exclusion Criteria
Patients treated with physician-modified endografts using the TREO (Terumo Aortic) device between June and December 2024	Patients treated with custom-made fenestrated endovascular aortic repair
Urgent aortic pathologies including aortic rupture, symptomatic aneurysm, or type Ia endoleak with sac growth	Patients treated with physician-modified endografts other than TREO during the study period
Patients ineligible for standard endovascular aortic repair or open surgical repair due to anatomical unsuitability or comorbidity	

**Table 2:** Patient baseline characteristics.

Baseline Characteristics	
Age	82 SD: 4.6
Sex (male)	3/5
Hypertension	5/5
Diabetes	1/5
Coronary artery disease	3/5
Atrial fibrillation	3/5
Nicotine	2/5
Previous abdominal surgery	1/5

**Table 3:** Treatment indications and previous aortic surgery.

Patient	Indication	Previous Endovascular Infrarenal Procedure	Date of the Previous Endovascular Infrarenal Aortic Procedure
**1**	Ruptured PAU ^1^	0	
**2**	Covered rupture caused by type Ia endoleak	1	03/2015
**3**	Symptomatic AAA ^2^ caused by type Ia endoleak	1	07/2018
**4**	Ruptured infectious PAU	0	
**5**	Symptomatic pararenal AAA	0	

^1^ penetrating aortic ulcer; ^2^ abdominal aortic aneurysm.

**Table 4:** Intraprocedural details.

Patient	Number of Fenestrations	Surgery Time (min)	CSF ^1^-Drain	Modified Prosthesis	Bridging Stent Graft Details
**1**	3	180	0	22 × 70 Cuff	Gore VBX 5 × 29 mm, 6 × 29 mm and 7 × 29 mm
**2**	3	110	1	26 × 70 Cuff	Gore VBX 5 × 29 mm, 5 × 29 mm and 7 × 29 mm
**3**	2	120	0	28 × 70 Cuff	Bentley BeGraft peripheral 5 × 18 mm and 6 × 28 mm
**4**	1	110	0	30 × 80 × 14 bifurcated	Advanta V12 8 × 38 mm
**5**	4	350	0	28 × 100 × 14 bifurcated	Gore Viabahn VBX 5 × 29 mm, 5 × 29 mm, Advanta V 12 6 × 22 mm and 7 × 32 mm

^1^ cerebrospinal fluid.

**Table 5:** Bridging stent graft implantation time (min).

Patient	LRA ^1^	RRA ^2^	SMA ^3^	CT ^4^
**1**	5	5	3	0
**2**	11	5	3	0
**3**	4	5	0	0
**4**	0	0	6	0
**5**	10	2	13	10

^1^ left renal artery; ^2^ right renal artery; ^3^ superior mesenteric artery; ^4^ celiac trunk.

**Table 6:** Patient outcomes.

Patient	Any Type Endoelak	Endoleak I or III	Reintervention	In-Hospital Death	ICU ^1^ Stay in Hours	In-Hospital Stay in Days
**1**	0	0	0	0	24	6
**2**	0	0	0	0	18	6
**3**	0	0	0	0	40	12
**4**	0	0	0	0	24	8
**5**	1	1	1	0	24	20

^1^ intensive care unit.

## Data Availability

The raw data supporting the conclusions of this article will be made available by the authors on request.

## Supplementary Materials

The following supporting information can be downloaded at: https://www.mdpi.com/article/10.3390/jcm14144830/s1, Video S1: How to perform the fenestrated PMEG with the TREO device; Video S2: Resheathing of a Zenith Alpha EVAR system with the dedicated TAVR crimping device.

## Abbreviations

The following abbreviations are used in this manuscript:

AAA	Abdominal aortic aneurysm
AF	AF atrial fibrillation
BEVAR	Branched endovascular aortic repair
BSG	Bridging stent graft
CAD	Coronary artery disease
CTA	Computed tomography angiography
CSF	Cerebrospinal fluid
EVAR	Endovascular aortic repair
FEVAR	Fenestrated endovascular aortic repair
ICU	Intensive care unit
LRA	Left renal artery
OAR	Open aortic repair
PAU	Penetrating aortic ulcer
PMEG	Physician-modified endograft
RRA	Right renal artery
SMA	Superior mesenteric artery
STROBE	Strengthening the Reporting of Observational Studies in Epidemiology
TAVR	Transaortic valve repair
